# Comparison of Total Intravenous Anesthesia Versus Volatile Anesthesia on Arrhythmia Inducibility and Clinical Outcomes During Catheter Ablation for Ventricular Tachycardia

**DOI:** 10.1002/joa3.70218

**Published:** 2025-11-18

**Authors:** Jason Hui, Samual Turnbull, Ashwin Bhaskaran, Saurabh Kumar, Stefan Dieleman

**Affiliations:** ^1^ Sydney Medical School University of Sydney Sydney New South Wales Australia; ^2^ Westmead Applied Research Centre University of Sydney Sydney New South Wales Australia; ^3^ Department of Cardiology Westmead Hospital Westmead New South Wales Australia; ^4^ Department of Anaesthesia and Perioperative Medicine Westmead Hospital Westmead New South Wales Australia; ^5^ School of Medicine Western Sydney University Sydney Australia

**Keywords:** general anesthesia, inducibility, total intravenous anesthesia, ventricular tachycardia, volatile anesthetics

## Abstract

**Introduction:**

Catheter ablation is highly efficacious for the treatment of ventricular tachycardia (VT). In patients with structural heart disease, catheter ablation may be performed under general anesthesia (GA). There are limited data on the effect of anesthetic agents on VT inducibility. We compared VT inducibility using total intravenous anesthesia (TIVA) versus volatile anesthesia.

**Methods:**

In this retrospective observational study, patients who underwent catheter ablation for VT between January 2019 and May 2023 were included. Clinical data, procedural reports, and long‐term outcomes were collected from the electronic medical records. Patients were grouped based on the type of anesthetic agent used to maintain GA during the procedure.

**Results:**

There were 207 patients maintained under GA using TIVA and 56 patients using volatile anesthesia. One hundred and seventy‐five of the 207 (84.5%) patients in the TIVA group were inducible for VT compared to 38 of 56 (67.9%) in the volatile group (OR [95% CI]: 3.8 [1.4–10.4], *p* = 0.01). Male sex was identified as a potential factor associated with increased VT inducibility (OR [95% CI]: 4.7 [1.4–16.0], *p* = 0.01). TIVA patients had a shorter ventricular effective refractory period. However, there was no difference between either the number of extra stimuli needed to induce the VT, the proportion of VTs induced spontaneously, acute ablation success rate, or the incidence of VA recurrence.

**Conclusion:**

Use of volatile GA agents was associated with a higher incidence of VT non‐inducibility compared to TIVA. TIVA was associated with a lower risk of VA recurrence in follow‐up. The observed effect on VT inducibility could be explained by effects on ventricular effective refractory period.

## Graphical abstract

This study has identified that the maintenance of general anesthesia with TIVA as opposed to volatile anesthesia is associated with increased inducibility of ventricular tachycardia during catheter ablation procedures. 84.5% of TIVA patients were inducible compared to only 15.5%.s

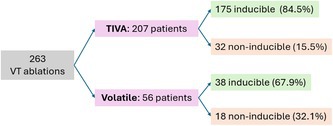

## 1 Introduction

Ventricular tachycardia (VT) is a life‐threatening arrhythmia and one of the most common causes of sudden cardiac death (SCD) [1, 2, 3]. VT frequently occurs in the presence of structural heart disease (SHD) predicated by re‐entry within and around scar caused by the underlying heart disease. Catheter ablation (CA) is a class I indication for VT refractory to anti‐arrhythmic drugs (AADs) and in patients with VT storm.

General anesthesia (GA) is often used to facilitate CA, with indications including (a) patient comfort; (b) protracted procedure duration; (c) need for multiple cardioversions; (d) potential for hemodynamic compromise during VT induction; and (e) need for ventilatory control to maintain adequate physiology and improve electroanatomic mapping accuracy as well as catheter stability during ablation. However, the agents used to induce and maintain anesthesia may suppress arrhythmia inducibility, partly through reduced sympathetic tone. The inability to induce VT during CA may limit procedural success, resulting in the need to default to empirical substrate ablation, inability to target culprit VT, and an increased risk of recurrence [4, 5]. Expert guidelines have, therefore, recommended avoiding very deep levels of anesthesia or sedation in order to increase the probability of successful arrhythmia induction and mapping [6].

GA for CA procedures is commonly delivered either intravenously with propofol or inhaled with a volatile agent such as sevoflurane. Previous studies have been conflicting when examining the effects of intravenous or volatile anesthetic agents on the inducibility of supraventricular tachyarrhythmias in children [7, 8, 9]. Furthermore, those studies focusing on volatile agents alone have found conflicting results on their ability to suppress supraventricular arrhythmias [10, 11]. There is a paucity of data comparing the impact of intravenous and volatile anesthetic agents on the arrhythmia inducibility during CA for VT.

This retrospective observational study aimed to compare the effects of intravenous anesthesia (propofol) with inhalational anesthesia (sevoflurane or desflurane) on the inducibility of VT during CA procedures. We hypothesized that inhalational anesthesia reduces VT inducibility more when compared to propofol.

## 2 Methods

### 2.1 Study Population

This was a retrospective observational study in patients who underwent CA under GA between January 2019 and May 2023 at Westmead Hospital, a quaternary VT referral center in Sydney, Australia. Patients who underwent CA for premature ventricular contractions, or who had CA under sedation only, were excluded. This study was approved by the Western Sydney Local Health District Research Ethics Committee.

### 2.2 Anesthesia Protocol

All patients underwent GA with either endotracheal intubation or a laryngeal mask airway to facilitate controlled mechanical ventilation. Routine monitoring (electrocardiogram, pulse oximetry, non‐invasive blood pressure, and end‐tidal respiratory gas monitoring) was used in all patients. An arterial line was inserted for continuous blood pressure monitoring at the discretion of the anesthetist.

Anesthesia was induced with a combination of propofol, an opioid (most commonly fentanyl and/or remifentanil), and a muscle relaxant in case of endotracheal intubation (most commonly rocuronium, vecuronium, or cisatracurium). Anesthesia was maintained with either a continuous propofol infusion or inhaled sevoflurane. The choice of anesthesia maintenance agents was based on the attending anesthetist's preference. The electrophysiologist was not involved in the decision‐making of intravenous versus inhalational anesthesia.

### 2.3 Electrophysiology Protocol

The VT induction protocol has been described previously [12]. Programmed electrical stimulation (PES) was performed from at least two right ventricular (RV) sites using a 400 ms drive train with up to four extra‐stimuli beginning at 300 ms, decrementing by 10 ms down to ventricular refractoriness. The endpoint of protocol was either ventricular refractoriness with the fourth extra‐stimulus or successful VT induction. If no VT was inducible, burst RV pacing down to refractoriness was performed at least five times. If VT was still non‐inducible, the above protocol was repeated with an isoprenaline infusion (2 mL bolus followed by an infusion that was gradually increased—in graded steps of 5, 10, 20, 30, and 40 micrograms/min, as hemodynamically tolerated), at peak infusion and in the “washout” phase.

### 2.4 Data Collection

Data were collected from the institutional electronic medical record, including patient demographics and baseline characteristics. All procedural and outcome data were collected from the Westmead Hospital VT database. For patients with an implantable cardioverter defibrillator (ICD), post‐ablation reprogramming was one to include VT treatment zones. The first zone was programmed below the rate of the slowest VT (with or without anti‐tachycardia pacing [ATP]) and the second zone was programmed at a minimum detection rate > 188 beats per minute programmed to deliver a shock (with or without ATP). All patients were enrolled in a remote monitoring service, managed by Westmead Hospital. Hospital medical records and outpatient clinic assessments were used to complete clinical follow‐up. Death was collected from medical records and the Social Security Death Index. Follow‐up was defined as the time from the final procedure to the last documented clinical review, device interrogation, or death.

### 2.5 Study Outcomes and Definitions

Patients were divided into two groups based on the GA agent used for maintenance, irrespective of what agent was used for anesthesia induction. The total intravenous anesthesia (TIVA) group was defined as maintenance with propofol, and the volatile group was defined as maintenance with either sevoflurane or desflurane. The choice of GA agent was at the discretion of the treating anesthetist.

The primary outcome was a binary endpoint of VT inducibility during CA. Secondary outcomes included the ease of VT inducibility, quantified by the number of ES needed, the S2 coupling interval (reflective of the ventricular effective refractory period [VERP]), and the proportion of VTs which were spontaneously induced without stimulation. Other secondary outcomes included VT tolerability, acute success, and long‐term recurrence of ventricular arrhythmias (VA).

VT tolerability was defined as VT not requiring termination due to hemodynamic instability. Acute success was defined as no inducible VT at the end of the procedure, provided that VT was inducible to begin with. Partial success was defined as the presence of at least one inducible nonclinical VT at the end of the procedure. Failure was defined as the inducibility of the clinical VT at the end of the procedure. Long‐term VA recurrence was defined as any VA lasting ≥ 30 s for patients without ICDs. In patients with ICDs, VA recurrence was defined as any appropriate device therapy, including anti‐tachycardia pacing or internal shock, or any VT lasting ≥ 30 s falling within the monitor‐only zone of the ICD.

### 2.6 Statistical Analysis

All continuous variables were expressed as mean ± SD, and categorical variables as proportions (95% confidence interval [CI]) with data stratified by GA maintenance group. Categorical variables were compared using Fisher's exact and chi‐squared tests. Where normally distributed, means were compared using independent samples *t*‐tests. Where data were non‐normally distributed, medians and distributions were compared using either Kruskal–Wallis *H* tests, or data was transformed for parametric testing. All univariable associations with *p* < 0.2 were considered candidate variables for multivariable analysis for predictors of the primary and secondary outcomes, which were performed using logistic regression. Recurrence of VA was analyzed using Cox proportional hazard analyses for recurrence‐free survival. Statistical significance was evaluated at the two‐sided *α* level of 0.05. All analyses were performed using SPSS version 28.0 for Windows (IBM Corp., Armonk, NY).

## 3 Results

**FIGURE 1:**
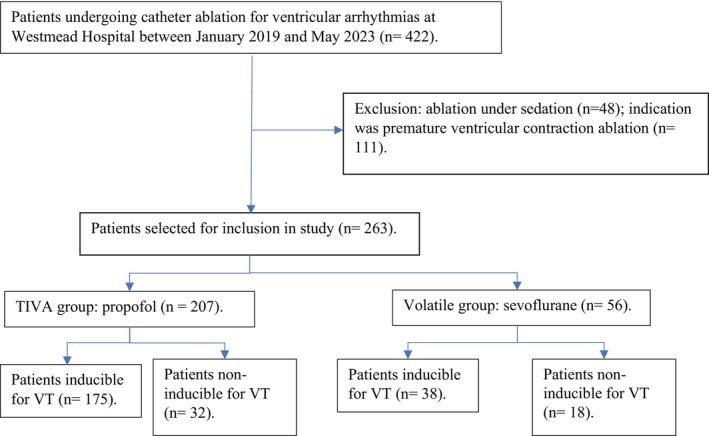
Flowchart of patient selection process for study.

A total of 422 patients underwent CA for ventricular arrhythmias at Westmead Hospital between January 2019 and June 2023 (Figure 1). After excluding 159 patients who underwent CA for PVCs, or who had procedural sedation alone, a final cohort of 263 patients who underwent CA for VT under GA was included for further analysis.

TIVA was used for GA maintenance in 207 (78.7%) patients and volatile anesthesia was used for 56 (21.3%). Characteristics for the two groups are summarized in Table 1. Apart from the presence of OSA, all other baseline characteristics were comparable between the two groups.

**TABLE 1:** Demographics of the study population.

Characteristic	Volatile (*n* = 56)	TIVA (*n* = 207)	*p*
Age (SD), y	61.8 (15.0)	60.11 (17.6)	0.524
Male sex—*n* (%)	51 (91.0)	169 (81.6)	0.105
BMI, mean (sd)	29.04 (5.2)	29.06 (6.3)	0.978
Comorbidities			
COPD—*n* (%)	6 (10.7)	20 (9.6)	1.000
Dyslipidemia—*n* (%)	22 (39.3)	79 (38.2)	1.000
HTN—*n* (%)	25 (44.6)	98 (47.3)	0.764
OSA—*n* (%)	2 (3.6)	32 (15.5)	**0.023**
PVD—*n* (%)	3 (5.4)	6 (2.9)	0.406
Stroke/TIA—*n* (%)	5 (8.9)	11 (5.3)	0.345
DM—*n* (%)	10 (17.9)	48 (23.2)	0.470
ICM—*n* (%)	24 (42.9)	85 (41.1)	0.879
NICM—*n* (%)	32 (57.1)	118 (57.0)	1.000
Idiopathic VT—*n* %	0 (0.0)	4 (2.4)	0.365
eGFR, mean (SD)	74.47 (19.6)	75.03 (17.8)	0.847
LVEF, mean (SD)	40.93 (12.9)	41.50 (15.9)	0.829
LVIDD, mean (SD)	56.97 (7.5)	56.21 (11.1)	0.700
Antiarrhythmic drug—no. (%)	46 (82.1)	168 (81.2)	1.000
Calcium channel blocker—no. (%)	0 (0.0)	9 (4.3)	0.212
Beta blocker—*n* (%)	31 (55.4)	121 (58.5)	0.761
Amiodarone—*n* (%)	18 (32.1)	67 (32.4)	1.000
Sotalol—*n* (%)	10 (17.9)	24 (11.6)	0.260
Other antiarrhythmic—*n* (%)	2 (3.6)	20 (9.7)	0.180
Implanted ICD—*n* (%)	43 (76.8)	172 (83.1)	0.278

*Note:* Bolded values represent statistical significance (*p* < 0.05).

Abbreviations: BMI, body mass index; COPD, chronic obstructive pulmonary disease; DM, type 2 diabetes mellitus; eGFR, effective glomerular filtration rate; HTN, hypertension; ICM, ischemic cardiomyopathy; LVEF, left ventricular ejection fraction; LVIDD, left ventricular internal diastolic diameter; NICM, non‐ischemic cardiomyopathy; OSA, obstructive sleep apnea; PVD, peripheral vascular disease; TIA, transient ischemic attack.

Despite a history of VA, 50 patients' VT could not be induced following induction of anesthesia in the baseline state before ablation using PES with up to four ES (Table 2). Notably, this was despite repeating the induction protocol with the use of high‐dose isoprenaline. Of these patients, 32 (64%) were in the TIVA group and 18 (36%) were in the volatile group (*p* = 0.01). VTs were inducible significantly more often in the TIVA group when compared to the volatile group (175/207 (84.5%) vs. 38/56 (67.9%); OR 2.59, 95% CI 1.32–5.09, *p* = 0.01).

**TABLE 2:** Potential factors affecting inducibility of ventricular tachycardia.

Characteristic	Non‐inducible (*n* = 50)	Inducible (*n* = 213)	Univariate OR (95% CI)	Univariate *p*	Multivariate OR (95% CI)	Multivariate *p*
Age (sd), y	55.68 (16.5)	61.58 (17.0)	1.02 (1.00–1.04)	0.027	1.00 (0.96–1.03)	0.68
Male sex—*n* (%)	35 (70.0)	185 (86.9)	2.83 (1.37–5.84)	0.01	4.69 (1.37–15.98)	**0.01**
BMI (sd)	28.91 (6.4)	29.09 (6.0)	1.01 (0.95–1.06)	0.850		
Type of GA agent			2.59	0.005	3.80 (1.39–10.37)	**0.01**
Volatile—*n* (%)	18 (36.0)	38 (17.8)				
TIVA—*n* (%)	32 (64.0)	175 (82.2)				
ICM—*n* (%)	16 (32.0)	93 (43.7)	1.65 (0.86–3.16)	0.132	0.407 (0.14–1.16)	0.09
NICM—*n* (%)	32 (64.0)	118 (55.4)	0.70 (0.37–1.32)	0.269		
Idiopathic VT (%)	2 (4.0)	2 (1.0)	0.34 (0.06–2.11)	0.227		
VT storm—*n* (%)	12 (24.0)	76 (35.7)	1.76 (0.87–2.56)	0.115	0.77 (0.29–2.08)	0.61
Mechanism of VT			2.44	0.118		
Re‐entrant VT—*n* (%)	22 (14.7)	128 (85.3)				
Focal VT—*n* (%)	25 (22.2)	88 (77.8)				
HTN—*n* (%)	19 (38.0)	104 (48.8)	1.56 (0.83–2.93)	0.167	1.43 (0.50–4.08)	0.50
PVD—*n* (%)	1 (2.0)	8 (3.8)	1.91 (0.23–15.65)	0.539		
Stroke/TIA—no. (%)	4 (8.0)	12 (5.6)	0.69 (0.21–2.23)	0.529		
DM—*n* (%)	14 (28.0)	44 (20.7)	0.67 (0.33–1.35)	0.260		
eGFR (sd)	80.31 (14.2)	73.63 (18.7)	0.98 (0.96–1.00)	0.026	0.98 (0.95–1.00)	0.15
LVEF (sd)	43.94 (15.6)	40.87 (15.2)	0.99 (0.96–1.01)	0.292		
LVIDD (sd)	52.62 (12.03)	57.22 (9.87)	1.04 (1.00–1.09)	0.024	1.04 (0.99–1.09)	0.12
Any antiarrhythmic drug—*n* (%)	40 (80.0)	174 (81.7)	1.12 (0.51–2.42)	0.782	0.45 (0.10–2.06)	0.31
Ca Channel Blocker—*n* (%)	0 (0.0)	9 (4.2)	0.80 (0.76–0.85)	0.139		
Beta blocker—*n* (%)	28 (56.0)	124 (58.2)	1.10 (0.59–2.04)	0.775		
Amiodarone—*n* (%)	12 (24.0)	73 (34.3)	1.65 (0.81–3.35)	0.162	0.69 (0.26–1.85)	0.46
Sotalol—*n* (%)	7 (14.0)	27 (12.7)	0.89 (0.36–2.18)	0.802		
Other antiarrhythmic—*n* (%)	4 (8.0)	18 (8.5)	1.062 (0.34–3.29)	0.917		

*Note:* Bolded values represent statistical signicance (*p* < 0.05).

Abbreviations: BMI, body mass index; COPD, chronic obstructive pulmonary disease; DM, diabetes mellitus; eGFR, effective glomerular filtration rate; HTN, hypertension; ICM, ischemic cardiomyopathy; LVEF, left ventricular ejection fraction; LVIDD, left ventricular internal diastolic diameter; NICM, non‐ischemic cardiomyopathy; OSA, obstructive sleep apnea; PVD, peripheral vascular disease; TIA, transient ischemic attack.

On univariable analysis of the primary binary outcome VT inducibility, significant associations with male sex, reduced eGFR, and increased left ventricle internal diastolic diameter (LVIDD) were found (all *p* < 0.05). In multivariable analysis, the type of anesthesia remained a significant predictor of VT inducibility, where TIVA was associated with increased VT inducibility compared to volatile anesthesia (OR 3.80, 95% CI 1.39–10.37, *p* = 0.01). Male sex was the only other significant predictor of VT inducibility (OR 4.69, 95% CI 1.37–15.98, *p* = 0.01).

**FIGURE 2:**
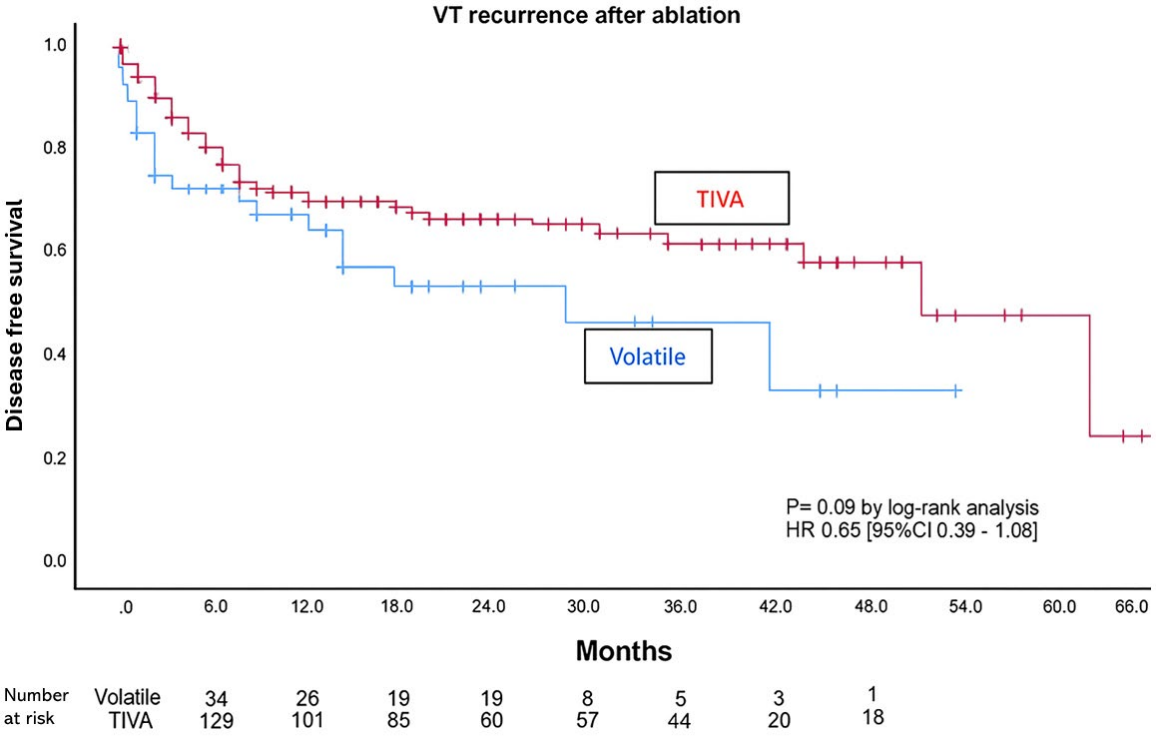
VT recurrence after ablation.

Secondary outcomes are reported in Table 3. There was no significant difference in the number of VTs induced, the number of ES required, or the proportion of VTs induced spontaneously between the TIVA and volatile groups. The mean cycle length and VT tolerability did not differ significantly between the two groups. The effective refractory period (S2 coupling interval) during PES was significantly lower in the TIVA group compared to the volatile group (252.70 ± 25.71 msec vs. 263.10 ± 36.62 msec) (*p* < 0.05). Radiofrequency ablation, X‐ray, and total procedure times were not different between TIVA and volatile groups. There was no statistically significant difference in acute procedural success rates between the two groups (Table 3, *p* = 0.26). At a median follow‐up of 20 months, there was a tendency toward a lower recurrence rate in TIVA (35% vs. 46%, *p* = 0.09 Figure 2).

**TABLE 3:** Secondary outcomes quantifying the degree of inducibility.

Characteristic	Volatile (*n* = 56)	TIVA (*n* = 207)	*p*
Number of ES—*n* (%)^a^			0.812
0 (spontaneous)	10 (13.5)	58 (13.7)	
1	9 (12.2)	36 (8.4)	
2	27 (36.5)	112 (26.4)	
3	18 (24.3)	93 (21.9)	
4	10 (13.5)	35 (29.5)	
Acute ablation success rate—*n* (%)			0.260
Failed	5 (8.9)	23 (11.1)	
Partial	7 (12.5)	46 (22.2)	
Success	44 (78.6)	138 (66.7)	
Inducible post isoprenaline—*n* (%)	3 (14.3)	9 (22.0)	0.343
S2 coupling interval (sd), ms	263.10 (36.6)	252.70 (25.7)	0.011
Mean cycle length (sd), ms	308.24 (67.2)	323.43 (75.0)	0.236
Procedure duration (sd)	237.93 (89.5)	234.06 (86.7)	0.293
Radiofrequency ablation time (sd), s	2621.74 (1638.1)	2538.03 (1795.2)	0.572
X‐ray time (sd), min	11.80 (7.4)	13.20 (7.0)	0.233
Mean number of inducible VT (sd)	2.16 (2.5)	2.67 (2.6)	0.179
Hemodynamically tolerated VT—*n* (%)	20 (48.8)	89 (51.1)	0.785

*Note:* Failed ablation (all arrhythmias were re‐inducible at the end of the procedure), partial success ablation (at least one inducible arrhythmia at the end of the procedure), successful ablation (no arrhythmias inducible at the end of the procedure).

Abbreviation: ES: extra stimulus.

^a^ This was calculated using the number of ES required for all inducible VTs in the study—not just the clinical VT.

## 4 Discussion

In this study, we have demonstrated several clinically relevant findings with respect to the potential influence of general anesthesia on VT inducibility during catheter ablation procedures. Anesthesia maintenance with a volatile agent was associated with a higher incidence of VT non‐inducibility in the baseline state before ablation, when compared with TIVA maintenance. Furthermore, TIVA maintenance was associated with a shorter ventricular refractoriness at the right ventricular apex during PES when compared to volatile anesthetic agents. No differences were demonstrated between the degree of inducibility with isoprenaline, the number of ES needed to induce VT, the proportion of spontaneously induced VTs, or the acute procedural success. Moreover, we noted a trend toward a higher recurrence rate of VT with the use of volatile anesthetic during follow‐up.

To our knowledge, this is the first study to explore the differential effects of type of anesthesia on the inducibility of VTs during CA procedures in adults. One previous study in a pediatric population has identified propofol to be associated with higher inducibility of supraventricular tachycardia (SVT) compared to volatile anesthetic agents [9]. This study showed that both time for induction to SVT and isoprenaline requirements were lower when using propofol was used compared to isoflurane. However, these results were not replicated in further studies [7, 8].

Previous studies have mechanistically explored the differential effects of anesthesia agents on cardiac electrical conduction in preclinical and human settings. Propofol has been shown to delay conduction through the sinoatrial [13, 14] and atrioventricular nodes [7, 14, 15, 16, 17], prolong the His‐ventricular interval [13, 14, 18] as well as shorten the ventricular effective refractory period and QT interval [19, 20]. In contrast, volatile agents have been shown to prolong the ventricular ERP and QT interval [7, 21, 22, 23]. Prolongation of the ventricular ERP is associated with a reduced inducibility of VT, which may explain greater non‐inducibility and the lower number of VTs induced in patients maintained with volatile anesthesia in our study [24]. Moreover, sevoflurane has been shown to have a strong lytic effect on sympathetic tone [25, 26] which can, in turn, prolong ventricular ERP [27, 28]. Sympathetic tone may also contribute to spontaneous PVCs that may incite re‐entrant VT [29]. Techniques to minimize sympathetic tone, such as cardiac sympathetic denervation and stellate ganglion blockade, have been shown to be effective in improving outcomes in patients with refractory VT [25, 26, 30, 31]. This suggests that a higher sympatholytic effect of volatile anesthetics compared to intravenous propofol could potentially explain the reduced VT inducibility seen in this study.

The practical implications of VT non‐inducibility during CA are significant. In patients with structural heart disease (SHD) and macroscopic myocardial scar, pre‐ablation non‐inducibility often necessitates an empirical approach to ablate all potential arrhythmogenic tissue identified through conventional substrate mapping techniques in sinus rhythm. Long‐term outcomes with this “substrate homogenization” approach have been modest, where recurrence can be driven by the fact that VT can occur, in some patients, from seemingly normal myocardium remote from scar [4, 5, 32]. In patients without SHD and minimal or no bipolar or unipolar scar, the complete non‐inducibility of any VT can mean that the patient is exposed unnecessarily to anesthesia, vascular access, and invasive cardiac procedural risk without any tangible benefit. The results of this study suggest that TIVA may be a preferable anesthetic maintenance agent in situations where inducibility of VT is paramount. Conversely, volatile agents can therefore be argued to be superior where reduced VT inducibility is favored, such as in patients undergoing non‐VT ablation procedures who are at high risk of VT.

### 4.1 Limitations

We acknowledge that our study has several limitations. First, this was a retrospective study, and therefore, there is the inherent risk that unmeasured confounders have not been accounted for and can thus lead to residual confounding. Second, there was discrepancy and imbalance between the number of cases using volatile anesthesia (*n* = 56) compared to TIVA (*n* = 207) which potentially weakens the statistical power of this study. Finally, due to limited availability of anesthesia monitoring data, our study has not been able to incorporate some of these data that could reflect the sympathetic suppressive effect of anesthesia in its analysis of VT inducibility. These include changes in heart rate before and after anesthesia induction, as well as the depth of anesthesia, which has been shown to reduce the inducibility of VT and was associated with increased VT recurrence [33].

## 5 Conclusion

The results of this retrospective study suggest that, compared to total intravenous anesthesia, volatile anesthesia is associated with a higher incidence of VT non‐inducibility during VT ablation procedures. Furthermore, volatile agents were associated with prolongation of the ERP, which may be a causal factor for this increased non‐inducibility. There was also a tendency toward lower arrhythmia recurrence with the use of TIVA. Further prospective, randomized controlled studies with a larger sample size are needed to validate these results.

## Conflicts of Interest

The authors declare no conflicts of interest.

## References

[1] B. A. Koplan and W. G. Stevenson , “Ventricular Tachycardia and Sudden Cardiac Death,” Mayo Clinic Proceedings 84, no. 3 (2009): 289–297, 10.1016/S0025-6196(11)61149-X. 19252119 PMC2664600

[2] S. M. Al‐Khatib , W. G. Stevenson , M. J. Ackerman , et al., “2017 AHA/ACC/HRS Guideline for Management of Patients With Ventricular Arrhythmias and the Prevention of Sudden Cardiac Death: Executive Summary: A Report of the American College of Cardiology/American Heart Association Task Force on Clinical Practice Guidelines and the Heart Rhythm Society,” Heart Rhythm 15, no. 10 (2018): e190–e252, 10.1016/j.hrthm.2017.10.035. 29097320

[3] C. Vaillancourt , M. Charette , I. G. Stiell , K. R. Phillips , and G. A. Wells , “Out‐Of‐Hospital Cardiac Arrest Surveillance in Canada: A Survey of National Resources,” CJEM: Canadian Journal of Emergency Medicine 12, no. 2 (2010): 119–127, 10.1017/s1481803500012148. 20219159

[4] L. Di Biase , P. Santangeli , D. J. Burkhardt , et al., “Endo‐Epicardial Homogenization of the Scar Versus Limited Substrate Ablation for the Treatment of Electrical Storms in Patients With Ischemic Cardiomyopathy,” Journal of the American College of Cardiology 60, no. 2 (2012): 132–141, 10.1016/j.jacc.2012.03.044. 22766340

[5] B. Nazer , C. Woods , T. Dewland , B. Moyers , N. Badhwar , and E. P. Gerstenfeld , “Importance of Ventricular Tachycardia Induction and Mapping for Patients Referred for Epicardial Ablation,” Pacing and Clinical Electrophysiology 38, no. 11 (2015): 1333–1342, 10.1111/pace.12703. 26228002

[6] E. M. Aliot , W. G. Stevenson , J. M. Almendral‐Garrote , et al., “EHRA/HRS Expert Consensus on Catheter Ablation of Ventricular Arrhythmias: Developed in a Partnership With the European Heart Rhythm Association (EHRA), a Registered Branch of the European Society of Cardiology (ESC), and the Heart Rhythm Society (HRS); in Collaboration With the American College of Cardiology (ACC) and the American Heart Association (AHA),” Heart Rhythm 6, no. 6 (2009): 886–933, 10.1016/j.hrthm.2009.04.030. 19467519

[7] T. O. Erb , R. J. Kanter , J. M. Hall , T. J. Gan , F. H. Kern , and S. R. Schulman , “Comparison of Electrophysiologic Effects of Propofol and Isoflurane‐Based Anesthetics in Children Undergoing Radiofrequency Catheter Ablation for Supraventricular Tachycardia,” Anesthesiology 96, no. 6 (2002): 1386–1394, 10.1097/00000542-200206000-00018. 12170051

[8] J. Lavoie , E. P. Walsh , F. A. Burrows , P. Laussen , J. A. Lulu , and D. D. Hansen , “Effects of Propofol or Isoflurane Anesthesia on Cardiac Conduction in Children Undergoing Radiofrequency Catheter Ablation for Tachydysrhythmias,” Anesthesiology 82, no. 4 (1995): 884–887, 10.1097/00000542-199504000-00010. 7717559

[9] I. T. Cohen , N. Furbush , and J. Moak , “Propofol Infusions for Radiofrequency Catheter Ablation for Supraventricular Tachycardia in Children,” Anesthesia & Analgesia 88 (1999): 291S, 10.1097/00000539-199902001-00288.

[10] M. S. Schaffer , A. M. Snyder , and J. E. Morrison , “An Assessment of Desflurane for Use During Cardiac Electrophysiological Study and Radiofrequency Ablation of Supraventricular Dysrhythmias in Children,” Paediatric Anaesthesia 10, no. 2 (2000): 155–159, 10.1046/j.1460-9592.2000.00465.x. 10736078

[11] J. D. Gallagher , “Electrophysiological Mechanisms for Ventricular Arrhythmias in Patients With Myocardial Ischemia: Anesthesiologic Considerations, Pt II,” Journal of Cardiothoracic and Vascular Anesthesia 11, no. 5 (1997): 641–656, 10.1016/s1053-0770(97)90021-5. 9263102

[12] T. Campbell , R. G. Bennett , K. Garikapati , et al., “Prognostic Significance of Extensive Versus Limited Induction Protocol During Catheter Ablation of Scar‐Related Ventricular Tachycardia,” Journal of Cardiovascular Electrophysiology 31, no. 11 (2020): 2909–2919, 10.1111/jce.14740. 32905634

[13] L. A. Pires , S. K. Huang , A. B. Wagshal , and R. S. Kulkarni , “Electrophysiological Effects of Propofol on the Normal Cardiac Conduction System,” Cardiology 87, no. 4 (1996): 319–324, 10.1159/000177113. 8793167

[14] M. H. Wu , M. J. Su , and S. S. Sun , “Age‐Related Propofol Effects on Electrophysiological Properties of Isolated Hearts,” Anesthesia and Analgesia 84, no. 5 (1997): 964–971, 10.1097/00000539-199705000-00004. 9141916

[15] D. F. Stowe , Z. J. Bosnjak , and J. P. Kampine , “Comparison of Etomidate, Ketamine, Midazolam, Propofol, and Thiopental on Function and Metabolism of Isolated Hearts,” Anesthesia and Analgesia 74, no. 4 (1992): 547–558, 10.1213/00000539-199204000-00015. 1554122

[16] C. Sochala , D. Deenen , A. Ville , and M. J. Govaerts , “Heart Block Following Propofol in a Child,” Paediatric Anaesthesia 9, no. 4 (1999): 349–351, 10.1046/j.1460-9592.1999.00373.x. 10411774

[17] M. F. Ganansia , T. P. Francois , X. Ormezzano , M. L. Pinaud , and J. Y. Lepage , “Atrioventricular Mobitz I Block During Propofol Anesthesia for Laparoscopic Tubal Ligation,” Anesthesia and Analgesia 69, no. 4 (1989): 524–525. 2571316

[18] M. Matsushima , S. Kimura , A. Kitaura , et al., “Propofol Suppresses the His‐Ventricular Conduction in Paediatric Patients,” Journal of Clinical Pharmacy and Therapeutics 46, no. 2 (2021): 433–439, 10.1111/jcpt.13302. 33098128 PMC7983984

[19] S. Paventi , A. Santevecchi , and R. Ranieri , “Effects of Sevoflurane Versus Propofol on QT Interval,” Minerva Anestesiologica 67, no. 9 (2001): 637–640. 11731753

[20] U. Higashijima , Y. Terao , T. Ichinomiya , K. Miura , M. Fukusaki , and K. Sumikawa , “A Comparison of the Effect on QT Interval Between Thiamylal and Propofol During Anaesthetic Induction,” Anaesthesia 65, no. 7 (2010): 679–683, 10.1111/j.1365-2044.2010.06341.x. 20528837

[21] M. Zaballos , B. Del Blanco , R. Sevilla , et al., “Differential Effects of Sevoflurane and Propofol on Swine Cardiac Conduction System,” Veterinary Anaesthesia and Analgesia 46, no. 3 (2019): 344–351, 10.1016/j.vaa.2018.11.007. 30833141

[22] H. Hashimoto , S. Imamura , K. Ikeda , and M. Nakashima , “Electrophysiologic Effects of Volatile Anesthetics, Sevoflurane and Halothane, in a Canine Myocardial Infarction Model,” Journal of Anesthesia 8, no. 1 (1994): 93–100, 10.1007/BF02482763. 28921208

[23] D. W. Han , K. Park , S. B. Jang , and S. E. Kern , “Modeling the Effect of Sevoflurane on Corrected QT Prolongation: A Pharmacodynamic Analysis,” Anesthesiology 113, no. 4 (2010): 806–811, 10.1097/ALN.0b013e3181f26d34. 20808206

[24] T. Kus , P. Costi , M. Dubuc , and M. Shenasa , “Prolongation of Ventricular Refractoriness by Class Ia Antiarrhythmic Drugs in the Prevention of Ventricular Tachycardia Induction,” American Heart Journal 120, no. 4 (1990): 855–863, 10.1016/0002-8703(90)90201-8. 2220538

[25] F. R. Assis , A. Sharma , R. Shah , et al., “Long‐Term Outcomes of Bilateral Cardiac Sympathetic Denervation for Refractory Ventricular Tachycardia,” Clinical Electrophysiology 7, no. 4 (2021): 463–470. 33812839 10.1016/j.jacep.2021.02.003

[26] M. Vaseghi , P. Barwad , F. J. Malavassi Corrales , et al., “Cardiac Sympathetic Denervation for Refractory Ventricular Arrhythmias,” Journal of the American College of Cardiology 69, no. 25 (2017): 3070–3080. 28641796 10.1016/j.jacc.2017.04.035 PMC6330109

[27] J. B. Martins and D. P. Zipes , “Effects of Sympathetic and Vagal Nerves on Recovery Properties of the Endocardium and Epicardium of the Canine Left Ventricle,” Circulation Research 46, no. 1 (1980): 100–110, 10.1161/01.res.46.1.100. 7349909

[28] H. Inoue and D. P. Zipes , “Results of Sympathetic Denervation in the Canine Heart: Supersensitivity That May Be Arrhythmogenic,” Circulation 75, no. 4 (1987): 877–887, 10.1161/01.cir.75.4.877. 3829345

[29] S. Franciosi , F. K. G. Perry , T. M. Roston , K. R. Armstrong , V. E. Claydon , and S. Sanatani , “The Role of the Autonomic Nervous System in Arrhythmias and Sudden Cardiac Death,” Autonomic Neuroscience 205 (2017): 1–11, 10.1016/j.autneu.2017.03.005. 28392310

[30] G. Murtaza , S. P. Sharma , K. Akella , et al., “Role of Cardiac Sympathetic Denervation in Ventricular Tachycardia: A Meta‐Analysis,” Pacing and Clinical Electrophysiology 43, no. 8 (2020): 828–837. 32460366 10.1111/pace.13968

[31] L. Meng , C.‐H. Tseng , K. Shivkumar , and O. Ajijola , “Efficacy of Stellate Ganglion Blockade in Managing Electrical Storm: A Systematic Review,” JACC: Clinical Electrophysiology 3, no. 9 (2017): 942–949. 29270467 10.1016/j.jacep.2017.06.006 PMC5734652

[32] R. D. Anderson , G. Lee , I. Trivic , et al., “Focal Ventricular Tachycardias in Structural Heart Disease: Prevalence, Characteristics, and Clinical Outcomes After Catheter Ablation,” JACC: Clinical Electrophysiology 6, no. 1 (2020): 56–69, 10.1016/j.jacep.2019.09.013. 31971907

[33] H. Dong , N. Li , and Z. Sun , “The Effect of Anesthesia Depth on Radiofrequency Catheter Ablation of Ventricular Tachycardia: A Retrospective Study,” BMC Anesthesiology 21, no. 1 (2021): 285, 10.1186/s12871-021-01503-6. 34781892 PMC8591932

